# The Antitumor Effects of α-Linolenic Acid

**DOI:** 10.3390/jpm14030260

**Published:** 2024-02-28

**Authors:** Huirong Yan, Senmiao Zhang, Li Yang, Mingjuan Jiang, Yujie Xin, Xuefei Liao, Yanling Li, Jianhong Lu

**Affiliations:** 1 Hunan Cancer Hospital/The Affiliated Cancer Hospital of Xiangya School of Medicine, Central South University, Changsha 410013, China; 2 Department of Medical Microbiology, School of Basic Medical Science, Central South University, Changsha 410078, China

**Keywords:** α-linolenic acid, polyunsaturated fatty acid, anticancer, tumor biology, drugs

## Abstract

α-linolenic acid (ALA), which is a member of the n-3 polyunsaturated fatty acid (n-3 PUFA) family, has often been ignored due to a lack of information. ALA has gradually attracted increased attention due to its nutritional and medicinal advantages. Studies have shown that ALA exerts beneficial effects on a variety of diseases, including cancer. In this review, we summarize the antitumor effects of ALA in the context of cell biology, including the inhibition of proliferation, the induction of apoptosis, the inhibition of metastasis and angiogenesis, and antioxidant effects. In addition, studies have shown that ALA can be used as a drug carrier or exert positive clinical effects when combined with drugs. Therefore, the use of ALA in clinical treatments is very promising and valuable.

## 1. Introduction

**Figure 1:**
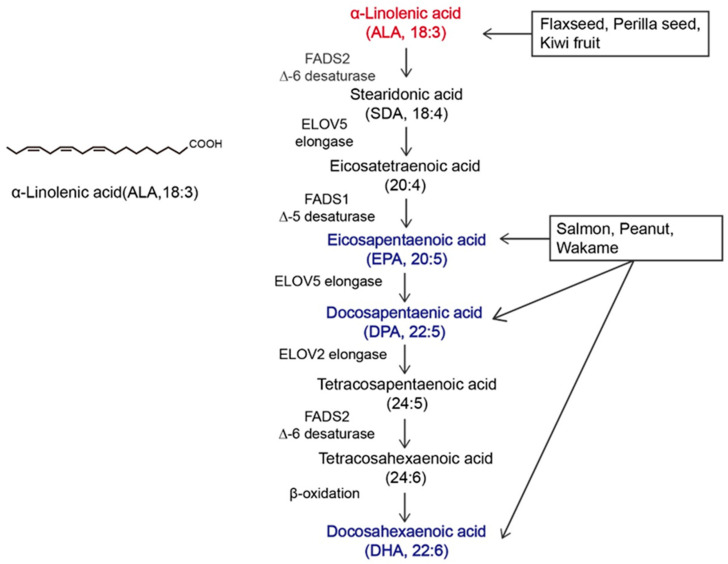
Overview of α-linolenic acid (ALA). Molecular structure of ALA and the in vivo metabolic pathway by which n-3 polyunsaturated fatty acids (n-3 PUFAs) are generated from ALA. The colors in the figure are intended to emphasize the members of n-3 PUFAs. The vertical arrow represents the metabolism of ALA to other n-3 PUFAs in vivo; Arrows in other directions represent different dietary sources of n-3 PUFAs.

α-linolenic acid (ALA) belongs to the family of n-3 polyunsaturated fatty acids (n-3 PUFAs) and contains a carbon–carbon double bond on the third carbon atom at the methyl end of the carbon chain. This family of essential fatty acids [1] also includes eicosapentaenoic acid (EPA) and docosahexaenoic acid (DHA). ALA is the synthetic precursor of these factors [2,3]. Studies have shown that ALA, DHA, and EPA can be converted into each other via metabolic pathways, as shown in Figure 1, but this conversion is inefficient [4,5]. In the past, attention to ALA was focused mainly on it as a precursor to DHA and EPA, while little was known about ALA itself [6,7]. The most common way to increase the levels of n-3 PUFAs in the body is through dietary intake. ALA can be acquired through the direct consumption of ALA-rich plants, such as flaxseed, perilla seed, chia seed, and rapeseed [8,9], as well as from various ALA preparations available on the market, such as ALA oil, soft capsules, and microcapsules [10]. As components of the phospholipid membrane [11], n-3 PUFAs play diverse roles, including cardiovascular disease prevention [12], anti-inflammatory effects [13], and anticancer effects [14]. However, the effects of ALA, DHA, and EPA on the human body are not consistent. Hemant Poudyal et al. [15] reported that ALA induced different physiological responses compared to DHA or EPA to alleviate the symptoms of metabolic syndrome. Jeong-Eun Choi et al. [16] showed that EPA and DHA exerted antidepressant effects on rats, while ALA did not exert antidepressant effects. Laura E Voorrips [17] showed that ALA was the only n-3 PUFA that was effective at reducing the risk of breast cancer (BC). Based on global medical and nutritional studies [9,18,19,20,21,22,23,24,25,26,27,28,29], ALA can regulate blood lipids, reduce blood viscosity, lower blood pressure, support weight loss, suppress allergic reactions, inhibit inflammation, affect diabetes and bone health, and inhibit cancer occurrence and metastasis. Therefore, it is necessary to distinguish ALA from other n-3 PUFAs.

According to the International Agency for Research on Cancer GLOBOCAN 2020 cancer incidence and mortality estimates, in 2020, there were 19.3 million new cancer cases and nearly 10 million deaths worldwide [30], except for melanoma cell cancer. As a populous country, China’s new cancer cases in 2020 accounted for 24% of the world’s new cancer cases [31]. Cancer has surpassed cardiovascular disease as the leading cause of death in China. A prominent feature of tumors is that their growth and proliferation are uncontrolled, and invasion and metastasis are the main problems facing current cancer treatment. Although the etiology of cancer is not yet fully understood, it can be roughly divided into two categories, endogenous and exogenous, and nutrients are the factors most closely related to daily life [32]. Nutrient intake can regulate the tumor microenvironment, thereby affecting cancer cell proliferation, apoptosis, and invasion. Current cancer treatment strategies, including surgery, radiotherapy, and chemotherapy, reduce the quality of life of patients, and diet has gradually become one of the most common treatment methods due to its high acceptance by patients and low toxicity and side effects [13,14]. Most of the initial dietary studies focused on limiting the proliferation of tumor cells by reducing the supply of major nutrients to tumors [33,34,35]. With further research, supplementation with specific nutrients, including histidine and mannose, has also become a strategy for the clinical treatment of cancer [36,37].

The n-3 PUFA family has attracted considerable attention for its anticancer effects and use as a dietary supplement. In this review, we examine the current literature, focusing on the role of ALA in anticancer effects and paying special attention to the mechanism of ALA in vivo and its effect on cancer-related characteristics. Additionally, we present instances of the use of ALA in combination with anticancer drugs. The goal of this review is to provide reference and inspiration for the further development and utilization of ALA.

## 2. Methods

We used PubMed, Google Scholar, the Wiley Online Library and the China National Knowledge Infrastructure to search for keywords such as ALA, antitumor effect, inhibition of metastasis, inhibition of proliferation, and combinations thereof to obtain relevant information. The effects of ALA on various tumors have also been studied. Information was collected until the submission of the review.

## 3. Anticancer Effects of ALA

**Figure 2:**
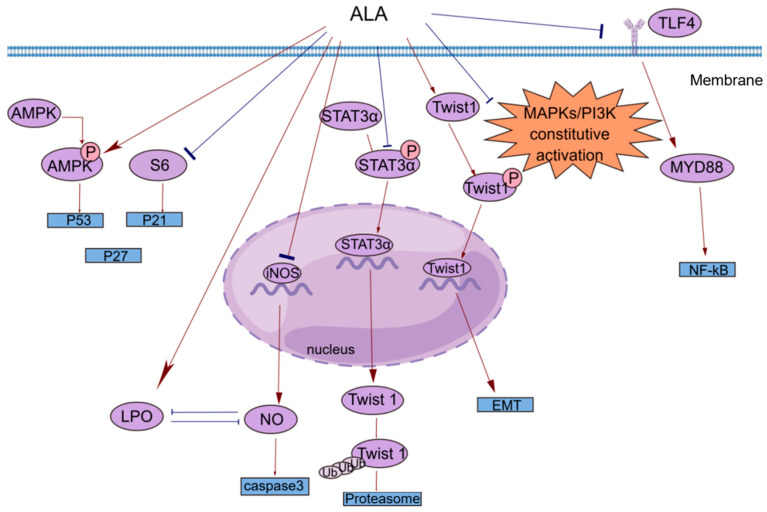
A brief summary of the molecular mechanism of the anticancer effects of ALA. ALA inhibits cell proliferation by regulating the AMPK/S6 axis. ALA can promote cell apoptosis by directly increasing intracellular lipid peroxidation (LPO) or indirectly reducing the accumulation of NO. ALA can suppress tumor metastasis by decreasing the mRNA expression of Twist1 and promoting the degradation of Twist1. The anti-inflammatory effects of ALA may be mediated by blocking the TLR4/MyD88/NF-κB cascade. This figure was constructed with FigDraw (ID: TOUPR3fbb8). There are two kinds of arrows, the flared arrows represent inhibition, and the other is facilitation.

Cancer has been a constant threat to human life since its identification, and even when it is treatable, it greatly reduces quality of life. Many studies have shown that ALA exerts significant anticancer effects on multiple cancers [38,39,40,41,42,43,44,45,46,47,48]. In Table 1, we list a subset of ALA-sensitive cancers, including prostate cancer, BC, hepatocellular carcinoma, colorectal cancer (CRC), and pancreatic cancer. In addition, ALA also exerts effects on many common gastrointestinal tumors and bladder cancer [49,50,51]. As shown in Figure 2, ALA exerts a variety of anticancer effects, including inhibiting proliferation, inducing apoptosis, suppressing tumor metastasis and angiogenesis, and exerting antioxidant effects. To provide a brief introduction to the anticancer effects of ALA, we systematically reviewed these effects, focusing on pharmacological actions and molecular mechanisms.

### 3.1. Inhibition of Proliferation

Cell proliferation plays a key role in life. Normal cell proliferation is critical for organismal growth, development, tissue repair, and metabolism. However, the abnormal expression of cancer-related genes in cells caused by various factors can lead to uncontrolled cell proliferation, which is an important part of cancer development.

Esophageal tumors can lead to dysphagia and strongly affect patient quality of life. In clinical practice, small and localized tumors are often surgically removed, but there is no way to perform surgery on larger or non-localized tumors. In addition, the resistance of esophageal cancer to chemotherapy has made the need for new therapies more urgent. Hyun-Seuk Moon’s team [53] reported that dietary ALA with or without oleic acid (OA) could inhibit the proliferation of the esophageal cancer cell lines OE19 and OE33 by regulating the AMPK/S6 axis to treat esophageal tumors. OA and ALA promoted the expression of tumor suppressor genes, such as p53, p21, and p27, by activating AMPK and/or decreasing the phosphorylation of S6. This provides a new idea for cancer treatment involving the consumption of ALA-containing foods for therapeutic purposes. Studies have shown that ALA alone or in combination with other drugs can inhibit cell proliferation in a variety of ways. Peroxisome proliferator-activated receptor-γ (PPAR-γ) is a nuclear receptor that regulates lipid homeostasis. As natural ligands of PPAR-γ, fatty acids can inhibit the growth of cancer cells by activating PPAR-γ [54]. Lijun Yang et al. [55] reported that ALA dose-dependently inhibited the proliferation of renal cell carcinoma (RCC) cells by significantly increasing PPAR-γ activity and gene expression and significantly inhibiting cyclooxygenase-2 (COX-2). COX-2 is an inducible enzyme involved in inflammation. Moreover, when ALA was combined with the PPAR-γ agonist rosiglitazone and the COX-2 inhibitor N-(3-pyridyl) indomethacinamide, its inhibitory effect on the proliferation of the human RCC cell line OS-RC-2 was further increased. ALA inhibited the transformation of cervical cancer cells by reducing the expression of the human papillomavirus oncoproteins E6 and E7, restoring the expression of the tumor suppressor proteins p53 and Rb, and reducing the expression of phosphorylated ERK1/2 and p38. Thus, cell proliferation was inhibited [56]. Consequently, it is possible and promising to use ALA in clinical practice to treat associated tumors by preventing tumor cell proliferation.

### 3.2. Induction of Apoptosis

Apoptosis is regulated by genes and is a type of programmed cell death. During embryonic development, certain cell populations undergo apoptosis to eliminate certain cells and complete organogenesis. However, mutagenesis by external factors can cause normal cells to become cancerous. The carcinogenesis of normal cells requires uncontrolled cell proliferation, the dysfunction of cell apoptosis, and the dysregulation of apoptotic regulators. In general, cancerous cells escape apoptosis by upregulating antiapoptotic factors and downregulating proapoptotic factors [57].

Most studies investigating the antiapoptotic effects of n-3 PUFAs have focused on major substances such as EPA and DHA, and little research has been conducted on ALA [58,59,60]. However, the functions of these substances are different. For example, one prospective study separated DHA, EPA, and ALA and found that ALA was the only n-3 PUFA that significantly reduced BC risk [17]. Interestingly, this phenomenon of local generalization is not uncommon. Similarly, Lilian U. Thompson et al. [61] reported that most studies of BC also ignored the effects of subtype and estrogen 17-b oestradiol (E2) levels, which are two key factors in BC growth and response to treatment. The authors subsequently investigated the effects of different doses of ALA on the growth of multiple BC cell lines and measured changes in total cellular phospholipid fatty acids to exclude the effects of DHA and EPA. The results showed that ALA-mediated apoptosis induction may be specific to the BC subtype. Under the same conditions, there was more apoptosis in basal cells than in luminal cells, and apoptosis in triple negative breast cancer (TNBC) cells was significantly higher than that in control cells. The extent of apoptosis was directly related to the level of ALA incorporated into the BC cells [61]. Caspases are a class of cysteine proteases that can mediate apoptosis. The apoptotic effect of ALA is closely related to its ability to increase lipid peroxidation [62]. An increase in lipid peroxides may increase the generation of free radicals, and reactive oxygen species (ROS) can directly activate mitochondrial permeability transition, leading to the loss of mitochondrial membrane potential. This results in cytochrome c (cyt c) release and caspase pathway activation. ALA reduced the mRNA expression of inducible nitric oxide synthase (iNOS) to reduce intracellular levels of NO, which can inhibit lipid peroxidation by scavenging free radicals from lipid peroxidation. ALA also inhibited iNOS-induced NO production in a peroxidation-dependent manner, further activating caspase 3 to induce apoptosis [62]. In addition, studies have shown that ALA can promote apoptosis in a variety of ways, such as by upregulating the expression of the proapoptotic gene Bax, downregulating the expression of the antiapoptotic gene Bcl-2, stabilizing hypoxia-inducible factor-1α (HIF-1α) and downregulating fatty acid synthase (FASN) to promote mitochondrial apoptosis [63,64]. This opens up multiple possibilities for the clinical use of ALA.

### 3.3. Anti-Inflammatory Response

The inflammatory response is a double-edged sword. When the normal balance of the body is disrupted, immune activity is increased and typically manifests as inflammation. However, the existence of inflammation itself and the changes in the microenvironment caused by inflammation cause certain pathological symptoms. Cancer patients are prone to secondary inflammatory diseases, and cancer may also develop in response to inflammation [65,66]. For example, patients with inflammatory bowel diseases such as ulcerative colitis and Crohn’s disease have an increased risk of CRC and a higher mortality rate than patients with sporadic CRC [67]. The main reason may be the recurrent chronic inflammatory response, which continuously damages the intestinal mucosa. The mucosa is in a state of long-term repair accompanied by intestinal microbial heterotopia and atypical hyperplasia, which can eventually lead to cancer [68]. ALA has been shown to exert powerful anti-inflammatory effects [69], and different epidemiological studies have shown that ALA is inversely correlated with plasma levels of inflammatory factors, including C-reactive protein (CRP), interleukin-6 (IL-6), interleukin-1β (IL-1β), interferon γ (IFN-γ), tumor necrosis factor-α (TNF-α), and E-selectin [25,26]. The potential anti-inflammatory mechanisms of ALA are diverse and include reducing the level of arachidonic acid (AA) in the blood and downregulating COX-2 [70].

A common clinical phenomenon in alcoholic hepatitis patients is the leakage of bacterial endotoxins through the damaged intestinal barrier into the portal vein. The endotoxins then bind to lipopolysaccharide-binding protein (LBP), triggering the TLR4/MyD88/NF-κB inflammatory cascade in the liver. ALA can inhibit the lipopolysaccharide (LPS)-induced inflammatory response by blocking this cascade [71]. N-6 polyunsaturated fatty acids, such as linolenic acid (LA), can be metabolized to AA, which is the precursor of many potent proinflammatory mediators, including prostaglandins (PGs) and leukotrienes (LTs) [72]. ALA intake can reduce blood levels of AA because ALA competes with LA for the enzyme delta-6-desaturase. Moreover, ALA is the preferred substrate of this enzyme. Increasing ALA intake can limit the conversion of LA to AA, thereby reducing the biosynthesis of proinflammatory eicosanoid acid and further exerting anticancer effects [52,73]. COX-2 enzymes are involved in the synthesis of proinflammatory prostaglandins, and one possible mechanism by which ALA inhibits cancer is that it inhibits inflammation through the downregulation of COX-2 [74]. NF-κB plays an important role in the inflammatory and immune responses. COX-2 is a downstream target of NF-κB activation. ALA suppresses tumors by reducing the expression of NF-κB and its target genes in tumor cells [56].

### 3.4. Inhibition of Tumor Metastasis

Tumors can spread in vivo to local normal tissues, to nearby lymph nodes, tissues, and organs, or to distant tissues through fluid transport, which is a feature that has made them a leading cause of cancer-related death [75]. Cancer metastasis can be divided into the following steps: tumor growth in situ, angiogenesis, epithelial–mesenchymal transition (EMT), invasion, intravasation, survival in the blood circulation, extravasation, dormancy, and metastatic tumor growth [76].

In vivo and in vitro experiments have shown that ALA inhibits metastasis in various cancers to varying degrees. Marianela Vara-Messler et al. [77] fed BALB/c mice a chia seed oil diet rich in ALA and corn oil rich in LA as a control and found that ALA could significantly reduce the incidence of BC and the number of metastatic lesions in mice. Mechanistically, ALA can alter signaling in BC cells by altering the cell membrane structure, which increases fatty acid unsaturation and exerts anticancer effects. In vitro experiments were performed to explore the mechanisms by which ALA inhibits cancer metastasis, including the EMT and tumor angiogenesis. Twist1 is required to initiate EMT and promote tumor metastasis and is regulated by signal transducer and activator of transcription 3α (STAT3α) and mitogen-activated protein kinases (MAPKs). Shih-Chung Wang et al. [39] studied the effect of ALA on Twist1 and Twist1-mediated TNBC cell migration and reported that ALA could reduce the mRNA expression of Twist1, reduce the accumulation of STAT3α in the nucleus, and reduce the protein and phosphorylation levels of Twist1. ALA promoted Twist1 degradation, thereby eliminating EMT and inhibiting Twist1-mediated migration in TNBC cells. One of the hallmark features of EMT is the loss of epithelial integrity due to the degradation of adherens junctions, which maintain epithelial cell contacts. A major driver of this degradation is proteolytic digestion by matrix metalloproteinases (MMPs). In tumors, MMP2 and MMP9 are involved in connective tissue degradation, tumor-induced angiogenesis, and cell migration. Another mechanism by which ALA inhibits tumor metastasis is by reducing vascular endothelial growth factor (VEGF), MMP-2 and MMP-9 protein expression [56]. As an important vasodilator, NO can stimulate VEGF production and participate in every step of VEGF-mediated tumor angiogenesis. NO can be generated by the enzyme iNOS in vivo. ALA not only reduces NO levels by reducing the mRNA expression of iNOS but also increases lipid peroxidation. The accumulation of peroxides increases the generation of free radicals; thus, the inactivation and reduction of NO decrease tumor angiogenesis and inhibit tumor metastasis [56,62].

### 3.5. Antioxidant Effect

Oxidative stress (OS) refers to the breakdown of the balance between ROS production and elimination in the body and is mainly characterized by the excessive production of highly reactive molecules such as ROS and reactive nitrogen species (RNS). OS is closely related to the occurrence and development of tumors [78,79]. Rashmi Deshpande et al. [62]’s study revealed that ALA could inhibit cancer by stimulating ROS production to induce apoptosis. However, this study was performed in a relatively simple in vitro experimental environment, and there are more complex and diverse mechanisms in vivo that can counteract this effect. Leslie Couedelo et al. [80] showed that ALA intake induced vitamin E depletion. Since vitamin E is a potent antioxidant, it can be used to capture the free radicals produced without producing OS. In addition, Jin Hyang Song and Teruo Miyazawa reported that excessive incorporation of n-3 PUFAs into cell membranes can have adverse effects by enhancing membrane sensitivity to lipid peroxidation (LPO) and inducing OS, but these studies were based on a high-fat diet [81]. In conclusion, when ALA is added to the diet, its effect on the body as a whole should be considered, and the amount of intake should also be considered.

Flaxseed oil (FO) is rich in ALA and is often used as a dietary supplement to treat cancer and improve health. Jyoti Sharma et al. [82] examined mice with skin cancer induced by 7, 12-dimethylbenzo-[a] thane (DMBA) combined with croton oil and showed that FO scavenged free radicals by increasing the levels of enzymatic and nonenzymatic antioxidants, including superoxide dismutase (SOD), catalase (CAT), glutathione peroxidase (GPx), and glutathione (GSH), in the skin and liver. SOD, CAT, and GPx are important antioxidant enzymes that work in concert to prevent excessive levels of intracellular ROS. The main role of SOD is to accelerate the dismutation of superoxide anions to hydrogen peroxide, after which CAT and GPx convert the resulting hydrogen peroxide into harmless substances [83]. The free radical scavenging effect of GSH is mediated by the active sulfhydryl-SH groups in its structure, which are easily dehydrogenated through oxidation. In addition, GSH can bind to GPx and requires different secondary enzymes and cofactors, including nicotinamide–adenine dinucleotide phosphate (NADPH), to exert its effects [82]. NADPH oxidase, which is the major source of ROS in the vasculature, is composed of a catalytic subunit and a regulatory cytosolic subunit. Hao Han [84] examined atherosclerosis and showed that FO could inhibit NADPH oxidase by reducing the mRNA and protein expression of the NADPH oxidase catalytic subunit, thereby regulating cytoplasmic subunit expression, reducing malondialdehyde levels and increasing GSH levels to exert antioxidant effects. ALA-rich FO is consumed mainly as a cooking oil, and there is undoubtedly a close association between this nutrient and the gut microbiota. Xiaoyan Sun et al. [85] used an FO diet to study the antioxidant capacity of colon epithelial cells in aged rats, and the results showed that the antioxidant levels of aged rats increased significantly after FO administration. Mechanistically, the intake of FO may have changed some intestinal bacteria. OS can play a role in the entire process of tumor development, and ALA intake can slow the toxic effects on the body when administered at different stages of tumor development.

### 3.6. Other Mechanisms

Unlike that of other unsaturated fatty acids, the protective effect of ALA on patients with nasopharyngeal carcinoma (NPC) was examined by Zhijie Fang et al. [86]. Based on differential expression analysis of ALA metabolism genes, the STRING database, Cytoscape software, and other tools were used to examine the mechanism by which ALA metabolism affects the prognosis of NPC patients. Immune infiltration analysis revealed that the risk score positively correlated with M2 and M0 macrophages and negatively correlated with neutrophils, plasma cells, follicular helper T cells, and resting dendritic cells. Low-risk patients were more sensitive to immunotherapy. In addition, pathway enrichment analysis revealed that the risk score was positively correlated with DNA repair and the G2/M checkpoint. Subhadeep Roy et al. [63] showed that ALA not only caused cell cycle arrest at the G2/M phase but also mediated tumor suppression by restoring cell structure, balancing metabolic abnormalities in rapidly growing cells, and inhibiting the hypoxic environment. Smita Eknath Desale et al. [87] showed that N9 microglia increased the phagocytosis of extracellular Tau and lysosomal-mediated Tau degradation after being exposed to ALA, suggesting that ALA can eliminate cancer cells by regulating lysosomal-mediated degradation. KCa3.1 channel activities are associated with abnormal cell proliferation, chronic inflammation, and autoimmune diseases. Aida Olivan-Viguera et al. [88] reported that ALA inhibited KCa3.1 channel and fibroblast mitosis. In other words, ALA may mediate tumor suppression by inhibiting mitosis and the KCa3.1 channel.

## 4. Drug and ALA Combinations for Anticancer Effects

Combination therapy is a common treatment strategy when a drug does not work well alone or when the toxic side effects of some drugs need to be neutralized. Combinations of drugs can enhance the antitumor effects of these agents and even reverse some known drug resistances [89]. The current research showed that combining ALA with several antitumor drugs enhanced the treatment efficacy and reduced the side effects of certain drugs [90,91,92,93].

At present, there are many clinical examples of the use of ALA combined with antitumor drugs for the benefit of patients. Trastuzumab (TRAS) is a first-line drug for the treatment of human epidermal growth factor receptor 2 (HER2)-overexpressing BC. But its efficacy as a single agent is only 12–26%, resistance develops within a year, and cardiotoxicity occurs in 5% of patients [90]. However, a low dose of FO combined with TRAS was more effective than TRAS alone at reducing the proliferation of tumor cells, thereby increasing the extent of tumor reduction. In other words, when combined with FO, a reduced dose of TRAS exerted the same effect as the original dose and had the advantages of fewer side effects and a longer duration of TRAS treatment [90]. Lilian U. Thompson et al. [91] reported that in the context of low circulating estrogen levels, FO combined with tamoxifen (TAM) effectively inhibited the growth of breast tumors by reducing cell proliferation and the expression of genes and proteins involved in signaling pathways mediated by estrogen receptor and growth factors. Cisplatin, which is a pioneering anticancer drug, has played a critical role in the history of cancer treatment. However, because of its lack of specificity when binding to DNA, the effect of cisplatin on cancer cells represents a typical “Pyrrhic victory”. The combination of ALA and cisplatin can increase antioxidant levels by increasing lipid peroxidation, thereby regulating the immune response, reducing the expression of the oncoprotein E6/E7, and increasing the expression of the tumor suppressor genes p53 and Rb, and a lower dose of cisplatin is needed to exert greater anticancer effects [92]. In addition, cisplatin can cause renal injury and increase oxidative stress in renal tissue, and ALA combined with cisplatin can protect the kidneys by reducing the activity of proinflammatory proteins, reducing the levels of iNOS and COX-2, and increasing the levels of GPx, SOD, and CAT [93]. Doxorubicin (Dox) is a broad-spectrum antibiotic that can exert antitumor effects by increasing the levels of ROS in cells. Oleg Shadyro et al. [94] reported that Dox combined with FO can not only reduce the intensity and volume of Pliss lymphosarcoma and Lewis lung adenocarcinoma but also inhibit Lewis lung adenocarcinoma metastasis. In addition, because PUFAs are components of the cell membrane, ALA may be used as a functional material for small molecule modification via coupling with antitumor drugs [95]. Tamara Zwain et al. [96] used ALA as a surface functionalization agent in combination with traditional docetaxel-coupled nanostructured lipid carriers (DTX-NLCs) and found that compared with bare DTX-NLCs, ALA could help DTX cross the blood–brain barrier and reach the target site. The combination of ALA and anticancer drugs can not only improve the efficacy of the drugs but also combat a variety of side effects, bringing new hope and ideas for the clinical treatment of cancer.

## 5. Conclusions and Perspectives

ALA is an n-3 PUFA with anti-inflammatory, antioxidant, and antitumor biological effects. Cancer is a serious threat to human health, and the current treatment methods for cancer still fail to meet the needs of patients. This review summarizes the molecular mechanism underlying the anticancer effects of ALA and its benefits when it is combined with anticancer drugs to provide a basis for the clinical development and use of ALA.

Diet therapy can exert anticancer effects by regulating the patient’s diet. It can prevent and control diseases by restricting diet and supplementing specific nutrients. Despite the continuous development of medical technology and the optimization of treatment methods, it is still not possible to completely control cancer. The prevention and treatment of cancer are the top priorities. ALA can be ingested through the diet and exerts good anticancer effects.

The addition of ALA to the daily diet can prevent cardiovascular disease and ameliorate inflammation and cancer. The anticancer effect of ALA is complex. First, ALA can inhibit the uncontrolled proliferation of cancer cells by regulating the AMPK/S6 axis, activating PPAR-γ, restoring the expression of the tumor suppressor proteins p53 and Rb, and regulating the cell cycle. Second, ALA can promote apoptosis by regulating mitochondrial membrane potential, activating caspases, upregulating proapoptotic factors, and downregulating antiapoptotic factors. Studies have shown a negative correlation between ALA and many inflammatory factors, including IL-6, IL-1β, PGs, LTs, and COX-2. In addition, cancer metastasis is an important cause of death in cancer patients. ALA can reduce cancer cell metastasis by altering the cell membrane structure to change cell signal transduction, inhibit tumor angiogenesis, and inhibit EMT. An appropriate dose of ALA can also reduce OS by regulating SOD, CAT, GPx, GSH, and NADPH oxidase to exert anticancer effects. Interestingly, ALA exerts its anticancer effects through dietary intake, which can alter or restore gut microbiota composition.

The delivery of drugs to tumor cells is a challenge that needs to be overcome. As a member of the n-3 PUFA family, ALA has the unique advantage of being integrated into the phospholipid membrane, which makes ALA a subject of interest for cancer treatment. The problems of drug resistance and the optimal treatment dose have troubled doctors and patients. ALA can be used as packaging to facilitate easier delivery of anticancer drugs to targets, which means that these drugs can function better at a lower dose and prolong the duration of drug use for patients to improve their survival. ALA can serve as a carrier to encapsulate anticancer drugs, and ALA alone can achieve tumor suppression.

Although the therapeutic effects of ALA have been studied to some extent, there is a knowledge gap regarding pharmacokinetics and pharmacodynamics in human subjects. Future studies should include comprehensive clinical trials to explore the entire process of ALA absorption and metabolism in vivo, determine the optimal dose, and evaluate the long-term effects of ALA. Furthermore, the role of ALA in some cancers is still controversial. There are many reasons for this gap, one of which is that the amount of ALA in most tissues, such as human blood, is very low and not easy to detect. This prompted us to further explore more optimized fatty acid detection methods. Although some studies have explored the effect of ALA in combination with drugs, additional in-depth studies are needed to understand the synergistic mechanism and whether ALA itself exerts anticancer effects by using an ALA delivery system based on nanotechnology. Future research can focus on the interaction of ALA with existing cancer treatments, including radiotherapy and chemotherapy; the possible underlying mechanism; the dose for long-term use; the possible effects and side effects; and the sustainability of its therapeutic benefits. In addition, the potential use of ALA to treat other diseases and the underlying mechanisms are important for improving the use of ALA. The high sensitivity of ALA to high temperatures, oxygen and metal ions, as well as the odor generated during transport and storage, limits its use in daily life. This will be an important area for future research to improve the use of ALA. By addressing these questions, future studies can greatly enhance our understanding of the therapeutic potential of ALA and optimize its clinical applications.

In conclusion, ALA has great potential and value in the treatment of cancer, and existing research has made some progress; however, its specific clinical applications still need to be further explored.

## Figures and Tables

**Table 1:** The mechanism of the antitumor effects of ALA.

Cancer	Effect	Effector Molecules	Change in Ex-Pression
PCa (prostate cancer) [52]	anti-inflammatory effect	PG/LTs	downregulation
BC (breast cancer) [38,39]	anti-inflammatory effect/inhibition of tumor metastasis	COX2/PGE2/Twist 1	downregulation
HCC (hepatocellular carcinoma) [40,41]	inhibition of proliferation	Farnesoid X receptor	upregulation
CRC (colorectal cancer) [42,43]	induction of apoptosis	caspase 3	downregulation
PCA (pancreatic cancer) [44]	anti-inflammatory effect	IL-1β/IL-6	downregulation

## Data Availability

Not applicable.
